# Thyroid Hormone-Regulated Wnt5a/Ror2 Signaling Is Essential for Dedifferentiation of Larval Epithelial Cells into Adult Stem Cells in the *Xenopus laevis* Intestine

**DOI:** 10.1371/journal.pone.0107611

**Published:** 2014-09-11

**Authors:** Atsuko Ishizuya-Oka, Mitsuko Kajita, Takashi Hasebe

**Affiliations:** 1 Department of Biology, Nippon Medical School, Kyonan-cho, Musashino, Tokyo, Japan; 2 Institute of Development and Aging Sciences, Nippon Medical School, Kosugi-cho, Kawasaki, Kanagawa, Japan; National Cancer Center, Japan

## Abstract

**Background and Aims:**

Amphibian intestinal remodeling, where thyroid hormone (T3) induces some larval epithelial cells to become adult stem cells analogous to the mammalian intestinal ones, serves as a unique model for studying how the adult stem cells are formed. To clarify its molecular mechanisms, we here investigated roles of non-canonical Wnt signaling in the larval-to-adult intestinal remodeling during *Xenopus laevis* metamorphosis.

**Methods/Findings:**

Our quantitative RT-PCR (qRT-PCR) and immunohistochemical analyses indicated that the expressions of Wnt5a and its receptors, frizzled 2 (Fzd2) and receptor tyrosine kinase-like orphan receptor 2 (Ror2) are up-regulated by T3 and are spatiotemporally correlated with adult epithelial development in the *X. laevis* intestine. Notably, changes in morphology of larval absorptive epithelial cells expressing Ror2 coincide well with formation of the adult stem cells during metamorphosis. In addition, by using organ cultures of the tadpole intestine, we have experimentally shown that addition of exogenous Wnt5a protein to the culture medium causes morphological changes in the larval epithelium expressing Ror2 even in the absence of T3. In contrast, in the presence of T3 where the adult stem cells are formed *in vitro*, inhibition of endogenous Wnt5a by an anti-Wnt5a antibody suppressed the epithelial morphological changes, leading to the failure of stem cell formation.

**Significance:**

Our findings strongly suggest that the adult stem cells originate from the larval absorptive cells expressing Ror2, which require Wnt5a/Ror2 signaling for their dedifferentiation accompanied by changes in cell morphology.

## Introduction

Adult organ-specific stem cells play critical roles in homeostasis and regeneration of vertebrate organs throughout adulthood. Especially, in the intestinal epithelium which undergoes rapid renewal, the adult stem cells are essential for life support, and their dysregulation results in development of various diseases including carcinogenesis. Recently, a growing body of evidence indicates important roles of canonical Wnt and Notch signaling pathways in maintenance of the stem cells in the adult mammalian intestine [1], [2]. However, it still remains poorly understood how the adult stem cells and their niche are formed during postembryonic development in any vertebrate. Its clarification at the molecular level should be not only interesting from the viewpoint of developmental and stem cell biology but also useful for regenerative medicine.

During amphibian metamorphosis, most of the larval organs undergo dramatic remodeling to adapt from aquatic to terrestrial life [3], [4]. In the *X. laevis* small intestine, we and others previously showed that the larval epithelium mostly undergoes apoptosis, whereas the adult epithelium develops from a small number of undifferentiated cells [5]–[7]. These undifferentiated cells become histologically detectable as small roundish islets between the larval epithelium and the connective tissue around NF stage 60 (early stage of metamorphic climax) [8]. The islets, which consist of a single or few cells at first, rapidly grow in size by active proliferation, invaginate into the connective tissue, and then differentiate into the single layer of adult epithelium as morphogenesis of intestinal folds proceeds. The adult epithelium after metamorphosis is rapidly renewed along the trough-crest axis of the intestinal folds [9] similar to that along the crypt-villus axis of the adult mammalian intestine [10], [11]. These chronological observations imply that the small islets of *X. laevis* intestine around stage 60 include the adult stem cells homologous to those in the mammalian intestine. In fact, increasing evidence indicates that mammalian intestinal stem cell markers such as Musashi-1 (Msi1) [12], [13] and leucine-rich repeat-containing G protein-coupled receptor 5 (Lgr5) [14]–[16] are specifically expressed in the islets of *X. laevis* intestine at and after stage 60 [17], [18]. Thus, this amphibian model offers a valuable opportunity to understand how the adult stem cells and their niche are formed during normal development. The key advantage of this amphibian model is that the whole process of the larval-to-adult intestinal remodeling including the adult stem cell formation can be experimentally reproduced both *in vivo* and *in vitro* by 3,5,3′-triiodothyronine (T3) [19], [20], a well-known causative agent of amphibian metamorphosis [3], [21], [22]. In particular, in the *X. laevis* intestine, numerous T3 response genes have been recently identified by microarray analyses [23]–[25] and provide us powerful clues to clarify molecular mechanisms underlying formation of the adult stem cells and their niche.

We have previously shown by tissue recombinant experiments *in vitro* that the adult stem cells originate from the larval epithelium of the *X. laevis* intestine at stage 57 before metamorphic climax [26]. Since the larval epithelium at this stage is fully differentiated as a simple columnar epithelium mainly consisting of absorptive epithelial cells, goblet cells, and enteroendocrine cells [27] and does not include any undifferentiated roundish cells expressing the stem cell markers [19], [20], it should be concluded that at least partly differentiated intestinal epithelial cells become the adult stem cells around stage 60 [26]. If so, the following questions arise: (1) what type of cells in the simple columnar larval epithelium have a potency to become the adult stem cells? (2) how do such columnar cells dedifferentiate into roundish stem cells and invaginate into the connective tissue by changing their morphology? To address these issues, we here focused on a non-canonical Wnt/planar cell polarity (PCP) pathway which has been reported to regulate cell polarity or migration in several organs [28], [29] including the mammalian embryonic gut [30]. Among T3 response genes identified so far in the *X. laevis* intestine, there are Wnt5a and its receptors, frizzled 2 (Fzd2) and receptor tyrosine kinase-like orphan receptor 2 (Ror2) [23], all of which are members of the PCP pathway. To know whether Wnt5a signaling is really involved in the amphibian stem cell formation, we first examined by quantitative RT-PCR (qRT-PCR) and immunohistochemistry the expressions of Wnt5a, Fzd2, and Ror2 in the *X. laevis* small intestine during metamorphosis. We found that their expression profiles correlate with the adult epithelial development but not with the larval epithelial degeneration. Especially, morphological changes of larval absorptive epithelial cells that express Ror2 coincide well with the formation of adult stem cells, suggesting important roles of Ror2 in this process. Next, by using Wnt5a protein and its function-blocking antibody in the organ culture of *X. laevis* intestine *in vitro*, we demonstrate that the Wnt5a/Ror2 signaling is essential for dedifferentiation of the larval epithelial cells into the adult stem cells.

## Materials and Methods

### Animals and Treatment

Tadpoles of the South African clawed frog (*Xenopus laevis*) at stages from 54 (premetamorphosis) to 66 (end of metamorphosis) were purchased and maintained from local suppliers and used throughout the experiments. Premetamorphic tadpoles at stage 54 were treated with 10 nM T3 (Sigma-Aldrich, St Louis, MO, USA) for 1 to 5 days. To inhibit protein synthesis, cycloheximide and anisomycin (Chx) dissolved in dimethyl sulfoxide (DMSO) were added to the rearing water of premetamorphic tadpoles at 20 and 25 mg/l, respectively [31]. DMSO was used for the control. After 1 h of Chx treatment, T3 was added at 50 nM, and the tadpoles were additionally treated for 6 h. At least 3 tadpoles were analyzed for each stage or day of T3-treatment. All experiments involving the *X. laevis* animals were approved by the Animal Use and Care Committee of Nippon Medical School.

### Quantitative Real-time Reverse Transcription-Polymerase Chain Reaction (qRT-PCR)

Total RNA was extracted from the small intestine of wild-type and T3-treated animals by using RNAiso reagent (Takara Bio, Shiga, Japan) followed by DNase treatment with DNA-free (Ambion, Austin, TX, USA) to remove any DNA contamination. The integrity of RNA was checked based on 18S and 28S ribosomal RNAs by electrophoresis. Total RNA was mixed with RNA-direct SYBR Green Real-time PCR Master Mix (Toyobo, Osaka, Japan), and then qRT-PCR was performed by using StepOnePlus Real-Time PCR System (Applied Biosystems, Carlsbad, CA, USA) according to the manufacturer's instructions. The primer pairs used are: 5′- TCGGTATCAAACAACCAGCC -3′ and 5′- CAATTTGCCCAAGTCTCTCC -3′ for Wnt5a, 5′- CCACAACTTTCATGGACTTAGC -3′ and 5′- AACATCATCAACAACCTGACG -3′ for Fzd2, 5′- CTGCTCTCTGGGAACTTTGG -3′ and 5′- GTCCCAGTGGGTCATTCAAG -3′ for Ror2. The level of specific mRNA was normalized against the level of rpL8 mRNA [32], [33] for each sample. Samples were analyzed in duplicate for 3 times. The specificity of the amplification was confirmed by the dissociation curve analysis and gel electrophoresis.

The qRT-PCR data during natural and T3-induced metamorphosis were analyzed with one-way ANOVA followed by the Tukey-Kramer post-hoc multiple comparison test. All qRT-PCR experiments were repeated with individual sets of RNA to ensure reproducibility (data not shown). On the other hand, the qRT-PCR data on Control vs T3 and those on Chx vs T3+Chx were analyzed by Student's T-test, respectively. When both were statistically significant (P<0.05), the gene was determined as the direct T3 response gene [34].

### Cloning of *X. laevis* genes and Western blot analysis

Using cDNA from stage 62 intestine as the template, Wnt5a and Ror2 genes were isolated by PCR with Wnt5a-255F (5′- GCACCATGAGAAAGAATCTGTGGAC -3′) and Wnt5a-1419R (5′- GGTCTGTGCTTGGAGTTCTACTTGC -3′), and Ror2-95F (5′ CTGCTCTCTGGGAACTTTGG -3′) and Ror2-3011R (5′- TGTGAAGATGTTTGTCCAAATTC -3′), respectively. The coding region of Wnt5a was isolated from the PCR product by the nested PCR with Wnt5a-CDS-F (5′- AATATTACCGGTGCCGCCACCATGAGAAAGAATCTGTGGACATTTC -3′) and Wnt5a-CDR-R-FLAG (5′- AATATTACTAGTCTACTTGTCGTCGTCATCCTTGTAGTCCTTGCATGCAAACTGGTCAACGACTTCG -3′). By this reaction, a FLAG tag was fused to the C-terminus of Wnt5a. The resulting cDNA was inserted into pT7Ts vector [35] and sequenced (pT7Ts_Wnt5a-FLAG). Using this as the template, Wnt5a protein was produced by TNT T7 Quick Coupled Transcription/Translation System (Promega, Madison, WI, USA) according to the manufacturer's instructions. The *in vitro*-translated (IVT) sample (5 µl) was analyzed by Western blotting using a goat polyclonal antibody against mouse Wnt5a (diluted 1∶500; R&D Systems, Minneapolis, MN, USA). The IVT sample reacted without the template was prepared as a negative control. The recombinant mouse Wnt5a (2 ng; R&D Systems) was loaded as a positive control.

The coding region of Ror2 fused with a FLAG tag at 5′ end was amplified using the PCR product by the nested PCR with Ror2-CDS-F (5′- AATATTACCGGTGCCGCCACCATGTCCAGGACCAGGAGCCAGAATGG -3′) and Ror2-CDS-R-FLAG (5′- TATTACTAGTCTACTTGTCGTCGTCATCCTTGTAGTCAGTCTCTGACTGAATATCTGTTG -3′). The resulting cDNA was inserted into pT7Ts vector and sequenced (pT7Ts_Ror2-FLAG). The mRNA encoding Ror2-FLAG was synthesized by mMessage-mMachine (Ambion) using the linearized pT7Ts_Ror2-FLAG as the template and purified with RNeasy Mini Kit (Qiagen, Valencia, CA, USA). Fertilized *Xenopus* eggs were prepared as previously described [35]. The synthesized mRNA was injected into one blastomere of 2- or 4-cell stage embryo at 1 ng/embryo. One day after injection, proteins were extracted from the embryos with IP buffer (20 mM HEPES, pH 7.5, 5 mM KCl, 1.5 mM MgCl2, 1 mM EGTA, 10 mM β-glycerophosphate, 50 mM NaCl, 0.1% IGEPAL, protease inhibitor cocktail (Roche Applied Science, Mannheim Germany). After centrifugation at 15000×g for 15 min at 4°C, the supernatant was subjected to immunoprecipitation (IP) using anti-FLAG-M2 agarose beads (Sigma-Aldrich). After the incubation overnight at 4°C, the beads were washed 3 times with IP buffer. Immunoprecipitates were eluted with 100 µg/ml 3x FLAG peptide (Sigma-Aldrich). The elutes were subjected to Western blotting using a rabbit polyclonal antibody against human Ror2 (diluted 1∶250; Sigma-Aldrich).

### Organ culture

Intestinal fragments were isolated from the anterior part of the small intestine in *X. laevis* tadpoles at stage 57 (before metamorphic climax) and then cultured as we previously described [36]. Briefly, tubular fragments were slit open lengthwise and were placed on membrane filters (type HAWP; Millipore, Billerica, MA, USA) on stainless steel grids in 60% Leibovitz-15 medium (Gibco-BRL, Grand Island, NY, USA) supplemented with 100 IU/ml of penicillin, 100 µg/ml of streptomycin, and 10% charcoal-treated fetal bovine serum (Gibco-BRL) (control medium). To induce metamorphosis, T3, insulin, and hydrocortisone (Sigma-Aldrich) were added to control medium at 20 nM, 5 µg/ml, and 0.5 µg/ml, respectively (T3-containing medium). Our previous studies have shown that, when the tadpole intestine is cultured in T3-containing medium for 5 days, T3-regulated gene expression leads to formation of the adult stem cells, whereas the intestine remains larval-type in control medium [17], [26], [37]. To examine the function of Wnt5a during the intestinal remodeling, some intestines were cultured in control medium added with recombinant mouse Wnt5a protein (4 µg/ml; R&D Systems), which is biologically active *in vitro* [38] and 90% identical to *X. laevis* counterpart. The others were cultured in T3-containing medium added with the goat anti-mouse Wnt5a antibody (8 µg/ml; R&D Systems), which inhibits the function of Wnt5a *in vitro* [38], on and after culture day 2 just before the up-regulation of endogenous Wnt5a mRNA expression. The anti-Wnt5a antibody whose epitope is 88% identical to the *X. laevis* counterpart really cross-reacted with the *X. laevis* Wnt5a protein with high specificity (Fig. S1). The culture medium was changed every other day for 5 days at 26°C.

### Immunohistochemistry

Intestinal fragments and cultured explants were fixed with 95% ethanol at 4°C for 4 h, embedded in paraffin, and cut at 5 µm. Some sections were immunostained with the following polyclonal antibodies at room temperature for 1 h: anti-Wnt5a antibody (diluted 1∶50; Lifespan Biosciences, Seattle, WA, USA), anti-Fzd2 antibody (diluted 1∶50; MBL, Woburn, MA, USA), and anti-Ror2 antibody (diluted 1∶200; Sigma-Aldrich). The epitopes of anti-Wnt5a and anti-Fzd2 antibodies are known to be highly identical (88% and 91%, respectively) to the *Xenopus* counterparts (manufacturers’ information). In addition, we confirmed that the anti-Ror2 antibody specifically recognized the *X. laevis* Ror2 protein by Western blot analysis (Fig. S2) These antibodies were then incubated with biotin-labeled anti-IgG and peroxidase-conjugated streptavidin (Nichirei, Tokyo, Japan) followed by 0.02% 3, 3′-diamino-benzidine-4HCl and 0.006% H_2_O_2_. In addition, to distinguish conveniently the adult stem/progenitor cells from the larval epithelial cells during the larval-to-adult epithelial replacement (stages 60–62), the other sections were stained with methyl green-pyronin Y (MG-PY) (Muto, Tokyo, Japan) for 5 min. Our previous studies have already shown that, during this period, the adult stem/progenitor cells are intensely stained red because of their RNA-rich cytoplasm, whereas the staining intensity of larval epithelial cells undergoing apoptosis become much weaker both *in vivo* and *in vitro* [6], [37], [39].

Furthermore, some other sections were double-immunostained at room temperature for 1 h with mixtures of the rabbit anti-Ror2 antibody and the mouse antibody against cytokeratin 19 (CK19) (1∶100; Novocastra, Newcastle, UK, USA) which is a predominant cytokeratin in the adult epithelium including the stem cells [40], Msi1 (1∶50; AbD Serotec, Oxford, UK, USA) [17], [40], or proliferating cell nuclear antigen (PCNA) (1∶100; Novocastra) for the detection of proliferating cells [6]. They were then incubated for 1 h with a mixture of Alexa Fluor 568-conjugated anti-rabbit IgG (1∶500; Molecular Probes, Eugene, OR) and 488-conjugated anti-mouse IgG antibodies (1∶500; Molecular Probes), counterstained with 4′, 6-diamidino-2-phenylindole, dihydrochloride (DAPI) (10 µg/ml; Dojindo, Kumamoto, Japan), and analyzed by fluorescence microscopy. Three to six fragments were examined for each developmental or experimental point. All control sections showed only background levels of signals, if any (data not shown).

## Results

### Up-regulated expressions of Wnt5a, Fzd2, and Ror2 mRNAs in the intestine during natural and T3-induced metamorphosis

**Figure 1:**
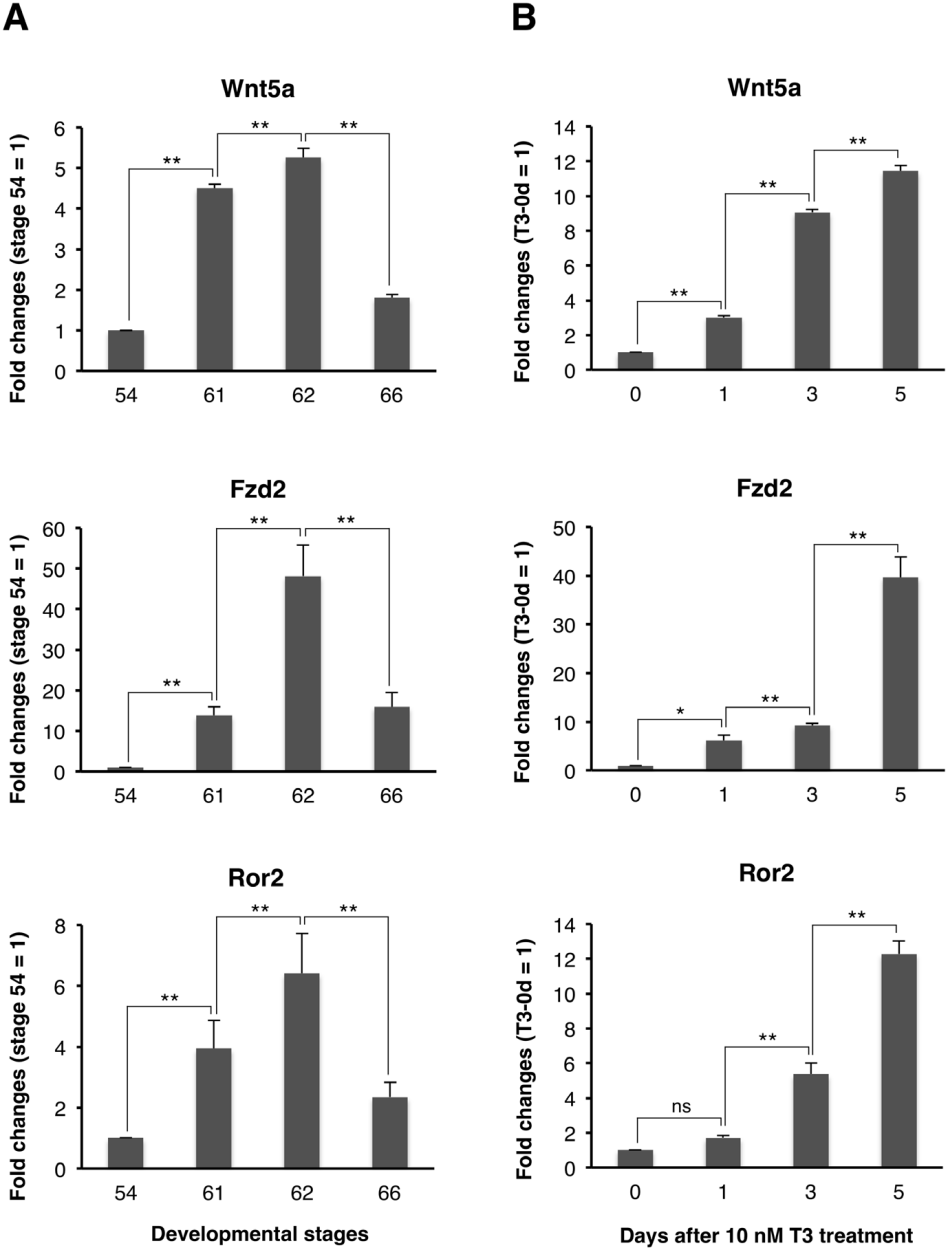
Up-regulation of Wnt5a, Fzd2, and Ror2 mRNAs in the *X. laevis* intestine during natural (A) and T3-induced metamorphosis (B). Total RNA was prepared from the intestine of tadpoles at indicated developmental stages (A) or stage 54-tadpoles after 10 nM T3 treatment (B), and was analyzed by qRT-PCR. The level of each mRNA is shown relative to the level of ribosomal protein L8 (rpL8) mRNA, with the values at stage 54 or day 0 of T3 treatment set to 1. Error bars represent the SEM (n = 4). Asterisks indicate that the transcription is significantly up- or down-regulated between the indicated stages or days after T3 treatment. *: P<0.05; **: P<0.01; ns: not significant.

To determine the temporal relationship between the expression of Wnt5a, Fzd2, and Ror2 mRNAs and the larval-to-adult intestinal remodeling, we first examined their expression in the *X. laevis* small intestine during natural metamorphosis by qRT-PCR. The expression of any mRNA remained very low at stage 54 (premetamorphosis; Fig. 1A), became abruptly up-regulated during stages 61–62 (early period of metamorphic climax), when the adult islets actively proliferate [6], and then was down-regulated toward stage 66 (end of metamorphosis).

**Figure 2:**
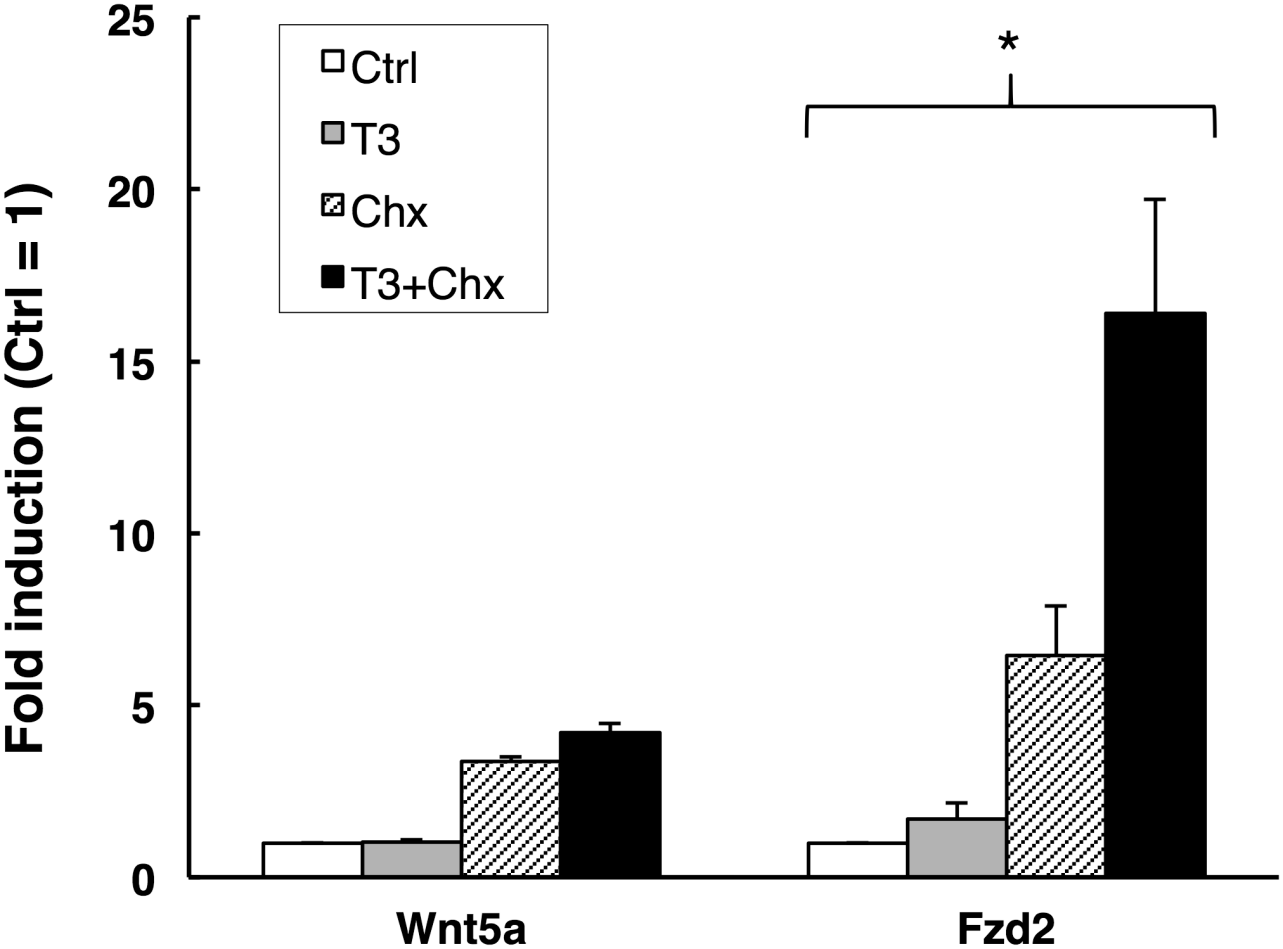
Fzd2 is a direct T3 response gene. Total RNA was isolated from the intestine of premetamorphic stage 54-tadpoles treated with DMSO (Control, white bars), 50 nM T3+DMSO (T3, gray bars), cycloheximide and anisomycin (Chx, shaded bars) and 50 nM T3+Chx (T3+Chx, black bars) for 6 h following the pretreatment with DMSO or Chx for 1 h. mRNA levels of indicated genes were examined by qRT-PCR. Control vs T3 and Chx vs T3+Chx were analyzed by Student's T-test, respectively. Error bars represent the SEM (n = 4). When the both were statistically significant, the gene was determined as the direct T3 response gene [34]. *: P<0.05.

Next, to examine whether T3 actually up-regulates the expressions of Wnt5a, Fzd2, and Ror2 mRNAs or not, we treated premetamorphic stage 54-*X. laevis* tadpoles with 10 nM T3 for 1 to 5 days. The expression of any mRNA in the small intestine was up-regulated similar to that during the early period of metamorphic climax and reached the highest level after 5 days of T3 treatment (Fig. 1B), when the islets develop [23]. These results strongly suggest the involvement of Wnt5a signaling pathway in adult epithelial development. In addition, qRT-PCR analysis showed that the expression of Wnt5a and Fzd2, but not Ror2, was significantly up-regulated only after 1 day of T3 treatment (Fig. 1B). This suggests that Wnt5a and Fzd2 genes may be the direct T3 response genes, whose expression is directly regulated by T3 via its receptor, independently of new protein synthesis. To verify this possibility, we added Chx to the rearing water of premetamorphic stage 54-tadpoles to block protein synthesis [31]. Treatment with Chx resulted in stronger induction of both Wnt5a and Fzd2 than that with T3, possibly because the protein synthesis inhibitors superinduce certain genes [41]. Importantly, even in the presence of Chx, T3 significantly up-regulated the expression of Fzd2 but not that of Wnt5a (Fig. 2). These results indicate that Fzd2 is likely a direct T3 response gene, whereas Wnt5a is an indirect one.

### Expression profiles of Wnt5a, Fzd2, and Ror2 proteins correlate with adult epithelial development

**Figure 3:**
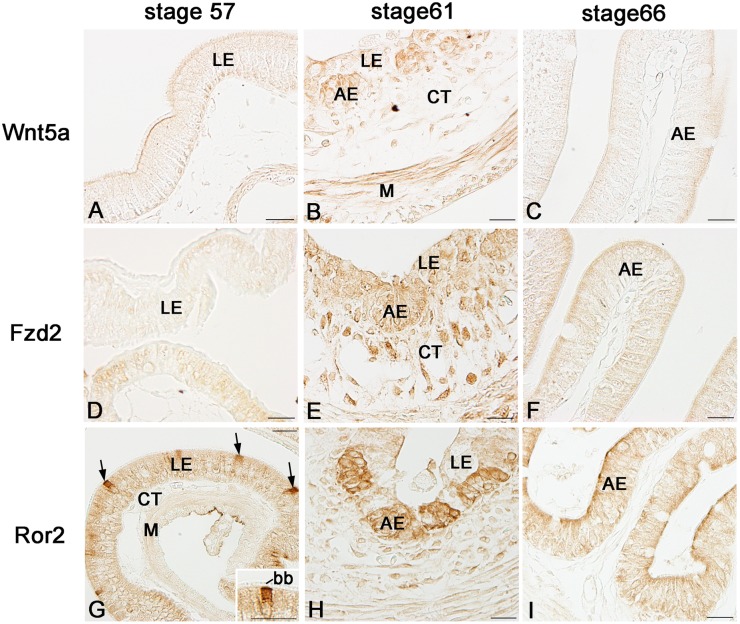
Expression profiles of Wnt5a, Fzd2, and Ror2 proteins in the small intestine during natural metamorphosis. Cross sections were immunostained with anti-Wnt5a (A–C), anti-Fzd2 (D–F), or anti-Ror2 (G–I). A, D, G: Stage 57. Cells positive for Ror2 (G; arrows) are scattered only in the larval epithelium (LE). They possess the brush border (bb) on the apical surface (Inset). B, E, H: Stage 61. Cells positive for Wnt5a (B) and Fzd2 (E) are broadly distributed in every tissue except for the degenerating larval epithelium, whereas a strong immunoreactivity for Ror2 is localized in islets of the adult epithelium (AE) (H). C, F, I: Stage 66. Immunoreactivity for each protein becomes weaker. CT: connective tissue; M: muscles; Scale bars: 20 µm.

**Figure 4:**
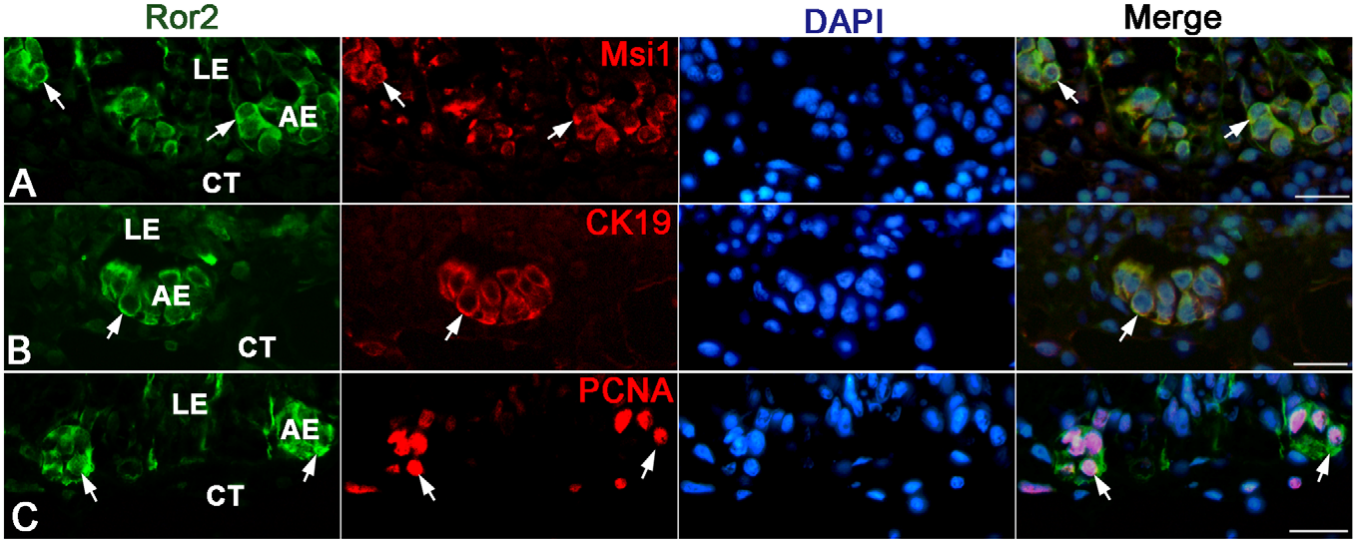
Ror2 expression is specific to adult epithelial stem/progenitor cells in the small intestine at stage 61. Cross sections were double-immunostained with anti-Ror2 (green) and anti-Msi1 (A; red), anti-CK19 (B; red), or anti-PCNA (C; red) antibodies, and counterstained with DAPI. Adult epithelial cells (AE) coexpress Ror2 and Msi1, CK19, or PCNA (arrows). CT: connective tissue; LE: larval epithelium. Scale bars: 20 µm.

We next examined by immunohistochemistry spatiotemporal correlations between the expression of Wnt5a, Fzd2, and Ror2 and adult epithelial development in the *X. laevis* small intestine. During stages 54–59, the tadpole intestine consists of the simple columnar epithelium, the immature connective tissue, and thin layers of inner and outer muscles. No cells immunoreactive for Wnt5a (Fig. 3A) nor Fzd2 (Fig. 3D) were definitely detected in any tissue. In contrast, a small number of cells immunoreactive for Ror2 were scattered as a single columnar cell in the entire larval epithelium (Fig. 3G). They were identified as absorptive epithelial cells, based on the long brush border on their apical surface. Thereafter, in agreement with the up-regulation of their mRNA expression shown by qRT-PCR, the immunoreactivity for Wnt5a, Fzd2, and Ror2 increased and attained its maximum level during stages 61–62. The immunoreactivity for Wnt5a (Fig. 3B) and Fzd2 (Fig. 3E) became broadly detected in every tissue except for the degenerating larval epithelium, although its intensity was different in different tissues. In contrast, Ror2 was highly expressed only in the islets growing in size (Fig. 3H). To characterize immunohistochemically these cells expressing Ror2, we performed double-immunofluorescence labeling in the *X. laevis* intestine at stage 61 with the antibodies against Ror2 and Msi1, CK19, or PCNA. The cells immunoreactive for Ror2 coincide well with those for Msi1 (Fig. 4A), those for CK19 (Fig. 4B), and those for PCNA (Fig. 4C), all of which are highly expressed in the adult epithelial stem/progenitor cells but not in the other larval cells during this period [6], [40]. Thereafter, as the islets differentiate into the simple columnar adult epithelium with the progress of intestinal fold-formation, the immunoreactivity for Wnt5a, Fzd2, and Ror2 gradually decreased (Fig. 3C, F, I), in agreement with the down-regulation of their mRNA expression shown by qRT-PCR.

### Larval absorptive cells expressing Ror2 dedifferentiate into adult stem cells

**Figure 5:**
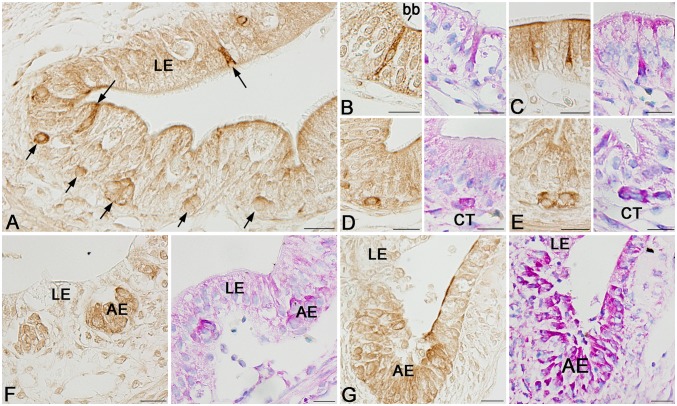
Ror2 expression correlates with formation of adult epithelial stem cells in the small intestine during stages 60–62. Cross sections were immunostained with anti-Ror2 (brown) or stained with MG-PY (blue and red). A–E: Stage 60. Epithelial cells positive for Ror2 show various morphologies and are scattered in the larval epithelium (LE) (A; arrows). They include simple columnar cells possessing the brush border (bb) (B), triangular-shaped cells (C), and roundish islet-like cells close to the connective tissue (CT) (D, E), all of which are strongly stained red with MG-PY. F: Stage 61. Ror2 expression is localized in islets of the adult epithelium (AE) stained red with MG-PY. G: Stage 62. Numerous adult epithelial cells are positive for Ror2, although the immunoreactivity per each cell gradually becomes weaker. Scale bars: 20 µm.

The expression profile of Ror2 described above raises the question whether the larval absorptive cells expressing Ror2 until stage 59 become the adult stem cells or not. To address this question, we examined more precisely the expression of Ror2 in the *X. laevis* intestine at stage 60, when the adult stem cells just appear. Interestingly, the epithelial cells immunoreactive for Ror2 at this stage revealed various morphologies from the simple columnar to roundish islet-like cells (Fig. 5A). To know whether these cells immunoreactive for Ror2 are larval cells destined to undergo apoptosis (larva-proper cells) or the other epithelial cells that become the adult stem cells (pre-adult stem cells), tissue sections close to those used for Ror2 immunohistochemistry were stained with MG-PY. Since MG-PY stains the adult stem/progenitor cells strongly red but does not the larva-proper cells at and after stage 60 [37], [39], these two-types of epithelial cells can be easily distinguished by the intensity of MG-PY staining at this stage. Obviously, the cells immunoreactive for Ror2 spatiotemporally agree well with the pre-adult stem cells stained strongly red with MG-PY. They include simple columnar cells similar to the larval absorptive cells expressing Ror2 until stage 59 (Fig. 5B), a single or few roundish cells just as the small islets localized between the larval epithelium and the connective tissue (Fig. 5D, E), and triangular-shaped cells intermediate between them (Fig. 5C). Then, during stages 61–62, the immunoreactivity for Ror2 was localized in typical islets consisting of the adult stem/progenitor cells, although its intensity per cell gradually became weaker as the islets grew into the connective tissue by active proliferation (Fig. 5F, G). These chronological observations strongly suggest that the larval absorptive cells expressing Ror2 is the origin of the adult stem cells and begin to dedifferentiate into the stem cells at stage 60, when the plasma T3 level becomes high [42] and thus up-regulates the expression of Wnt5a.

### Effects of Wnt5a/Ror2 signaling on adult stem cell formation *in vitro*

**Figure 6:**
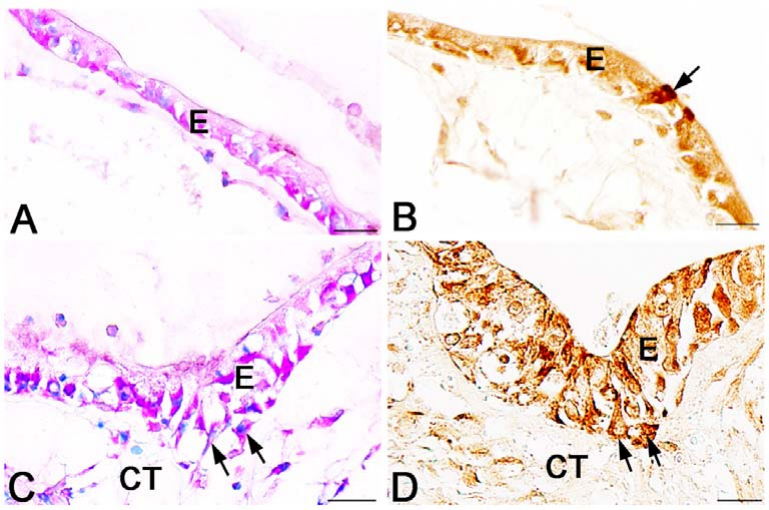
Effects of exogenous Wnt5a protein on tadpole intestines cultured for 5 days in the absence of T3. Cross sections were stained with MG-PY (A, C) or immunostained with anti-Ror2 (B, D). A, B: Control intestines. Cells positive for Ror2 are scattered in the simple columnar epithelium (B; arrow). C, D: Intestines cultured with the addition of Wnt5a. Epithelial cells positive for Ror2 change in morphology (arrows) and often invaginate into the connective tissue (CT). Scale bars: 20 µm.

**Figure 7:**
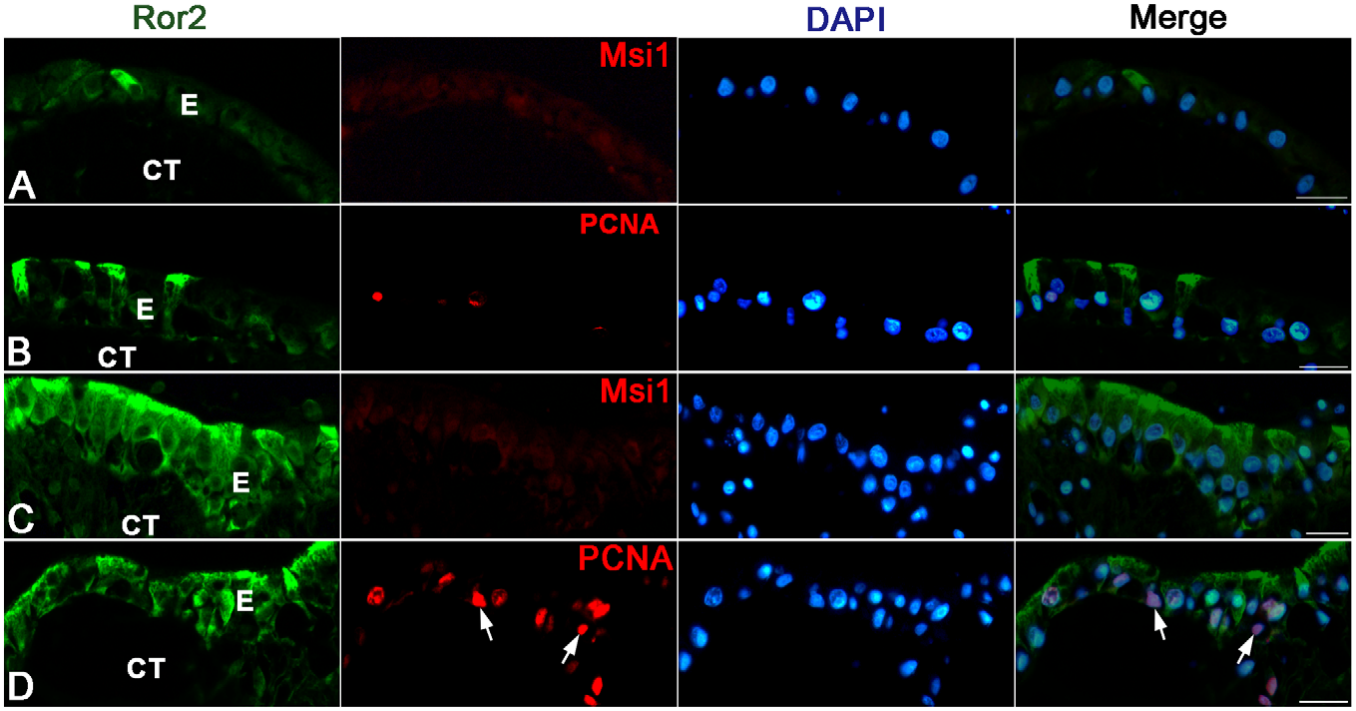
Wnt5a alone does not induce adult stem cells in tadpole intestines cultured for 5 days in the absence of T3. Cross sections were double-immunostained with anti-Ror2 (green) and anti-Msi1 (A, C; red) or anti-PCNA (B, D; red) antibodies, and counterstained with DAPI. A, B: Control intestines. The simple columnar epithelium (E) remains negative for Msi1 (A). Proliferating cells positive for PCNA are very few, if any (B). C, D: Intestines cultured with the addition of Wnt5a. Epithelial cells positive for Ror2 invaginate into the connective tissue (CT) but remain negative for Msi1 (C). Proliferating cells are more numerous than those in the control intestines and are also detectable in cells negative for Ror2 (D; arrows). Scale bars: 20 µm.

To clarify potential roles of Ror2 in the adult epithelial development, we examined effects of Wnt5a protein, the only ligand for Ror2 reported so far, on the larval epithelium expressing Ror2 by using an organ culture system previously established [33]. The intestine isolated from the *X. laevis* tadpoles at stage 57 remained larval-type throughout 5 days of culture in control medium lacking T3 (control intestine) (Fig. 6A). That is, just as the larval epithelium until stage 59 *in vivo*, the epithelium retained the simple columnar structure where cells immunoreactive for Ror2 were scattered (Fig. 6B). No adult stem cells immunoreactive for Msi1 were detected in any control intestine (Fig. 7A). Although proliferating cells were rarely detected, their distribution was independent of the Ror2 expression (Fig. 7B). In contrast, when Wnt5a protein was added to the control medium, the epithelial morphology significantly altered. Its basal surface became undulating here and there after culture day 2 and then, on day 5, some of the epithelial cells invaginated into the connective tissue (Fig. 6C), similar to the adult stem/progenitor cells during stages 60–61 *in vivo*. Such epithelial cells invaginating often showed the immunoreactivity for Ror2 (Fig. 6D), suggesting that Wnt5a signals cause epithelial morphological changes by binding to Ror2. Moreover, Wnt5a protein added to the medium promoted proliferative activity of the intestine on day 5 (Fig. 7D). However, the proliferating cells were broadly distributed in the entire epithelium, independently of the Ror2 expression (Fig. 7D), and no adult stem cells immunoreactive for Msi1 were detected in any intestine cultured with the addition of Wnt5a throughout the cultivation (Fig. 7C).

**Figure 8:**
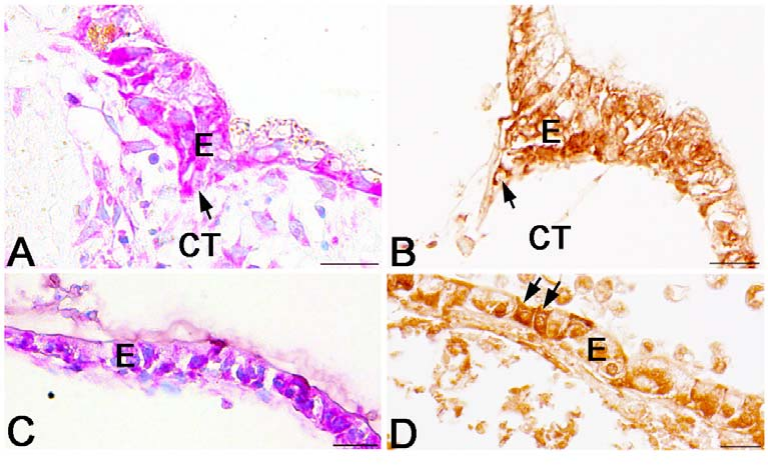
Effects of anti-Wnt5a antibody on T3-treated intestines cultured for 5 days. Cross sections were stained with MG-PY (A, C) or immunostained with anti-Ror2 (B, D). A, B: Intestines treated with 20 nM T3. Adult stem/progenitor cells stained strongly red with MG-PY and positive for Ror2 (A, B; arrow) invaginate into the connective tissue (CT). C, D: T3-treated intestines cultured with the addition of anti-Wnt5a antibody. The epithelium (E) remains a single layer, where cells positive for Ror2 are scattered (D; arrows). Scale bars: 20 µm.

**Figure 9:**
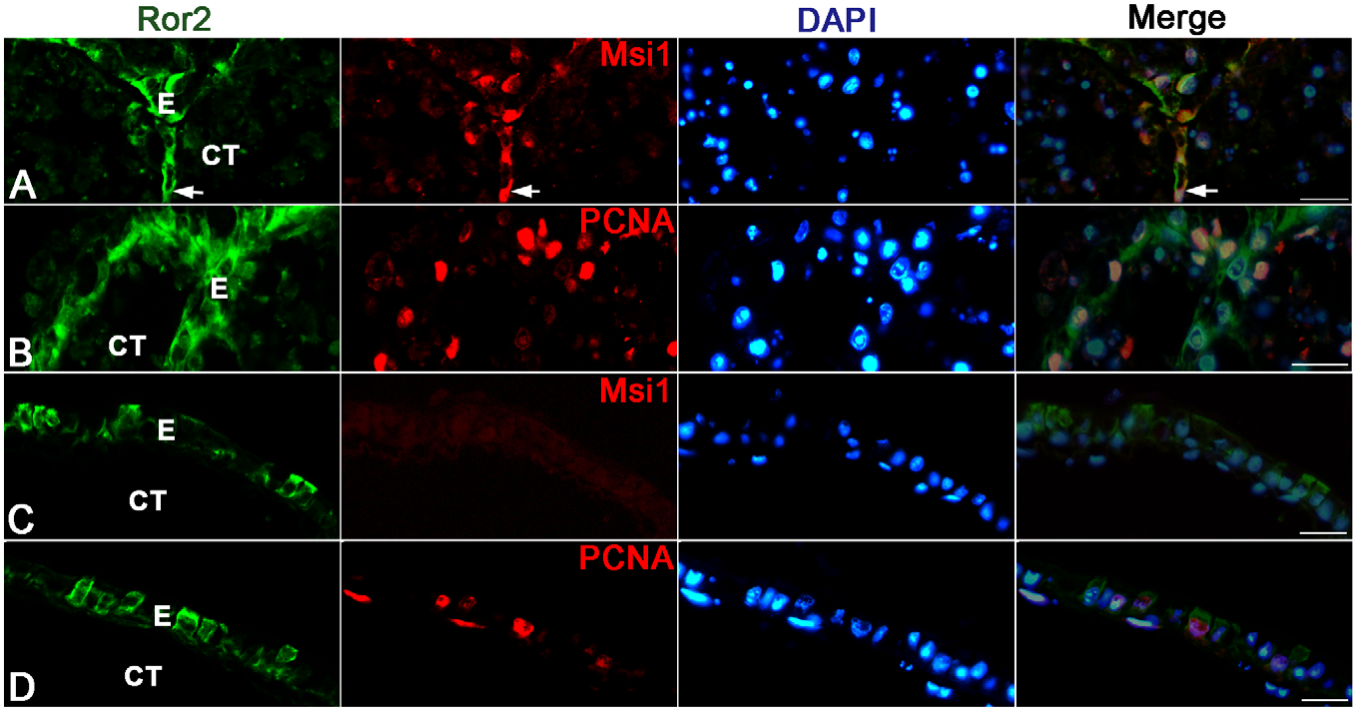
Anti-Wnt5a antibody inhibits adult stem cell formation in T3-treated intestines cultured for 5 days. Cross sections were double-immunostained with anti-Ror2 (green) and anti-Msi1 (A, C; red) or anti-PCNA (B, D; red) antibodies, and counterstained with DAPI. A, B: Intestines treated with 20 nM T3. Epithelial cells (E) invaginating into the connective tissue (CT) are double positive for Ror2 and Msi1 (A; arrow) and actively proliferate (B). C, D: T3-treated intestines cultured with the addition of anti-Wnt5a antibody. Epithelial cells positive for Ror2 remain negative for Msi1 (C). Proliferating cells are fewer than those in the T3-treated intestines in the absence of the antibody (D). Scale bars: 20 µm.

On the other hand, the tadpole intestine cultured in T3-containing medium (T3-treated intestine) underwent the larval-to-adult remodeling just like during natural metamorphosis as reported in our previous studies [17], [26]. Most of the larval epithelial cells underwent apoptosis, whereas the islets stained strongly red with MG-PY appeared and invaginated into the connective tissue by day 5 (Fig. 8A). The expression of Ror2 was localized in these islets (Fig. 8B). In addition, double-immunofluorescence labeling revealed that they consisted of adult stem/progenitor cells that expressed Msi1 (Fig. 9A) and actively proliferated (Fig. 9B). These epithelial changes are similar to those in the small intestine during stages 60–61 *in vivo*. In contrast, when the anti-Wnt5a antibody was added to the T3-containing medium to block the function of endogenous T3-induced Wnt5a protein, the epithelium mostly remained to be a single layer consisting of cuboidal or columnar cells (Fig. 8C). Although the epithelial cells stained strongly red with MG-PY (Fig. 8C) and the cells immunoreactive for Ror2 (Fig. 8D) were occasionally observed in the simple epithelium, no adult stem cells expressing Msi1 were detected in any intestine cultured in the presence of the antibody throughout the cultivation (Fig. 9C). While the larval epithelium underwent cell death like that in the T3-treated intestines, the antibody added to the T3-containing medium suppressed the proliferative activity of the intestine on day 5 (Fig. 9D), leading to a decrease in the total number of epithelial cells.

## Discussion

**Figure 10:**
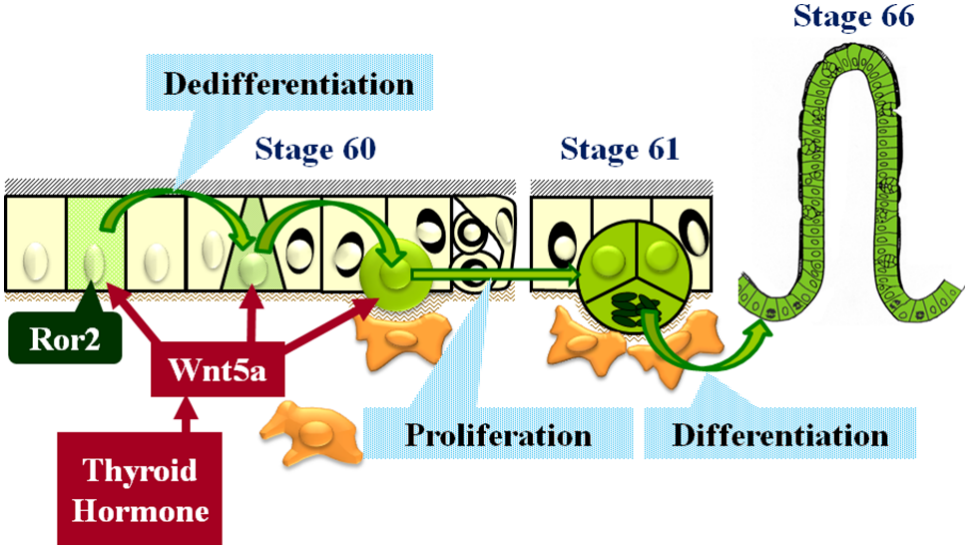
Schematic illustration showing potential roles of Wnt5a/Ror2 signaling in adult stem cell development in the *X. laevis* intestine. Larval absorptive cells expressing Ror2 are scattered in the simple columnar epithelium until stage 59. At stage 60, thyroid hormone up-regulates the expression of Wnt5a, whose protein binds to Ror2 of the absorptive cells and changes their morphology from simple columnar to roundish cells close to the connective tissue. This Wnt5a/Ror2 signaling is not sufficient but essential for epithelial dedifferentiation into the stem cells. In addition, Wnt5a promotes cell proliferation via receptors other than Ror2.

The *X. laevis* intestinal remodeling, where T3 induces adult stem cells homologous to mammalian ones, serves as a unique and valuable model for understanding how the adult stem cells and their niche are formed during postembryonic development [43]. Previously, our tissue recombinant experiments indicated that the adult stem cells are derived from the differentiated larval epithelium of the tadpole intestine before metamorphosis [26]. However, it still remains unknown what cells of the larval epithelium have a potency to become the adult stem cells, which can then generate the adult epithelium via several key signaling pathways such as Shh/Bmp-4 one [43], [44]. Here we have provided the first convincing evidence that only the larval absorptive cells that express Ror2 can dedifferentiate into the adult stem cells during metamorphosis and propose that T3-activated Wnt5a/Ror2 signaling plays a critical role in their dedifferentiation into the stem cells, possibly via a non-canonical Wnt/PCP pathway (Fig. 10).

### Adult stem cells originate from larval absorptive cells expressing Ror2

Ror2 is known to work as a receptor or coreceptor for the typical non-canonical Wnt ligand, Wnt5a [28], [29]. Although it is now well established that the canonical Wnt pathway is essential for maintenance and proliferation of the stem cells in the adult mammalian intestine [1], the roles of the non-canonical Wnt pathway in regulation of the adult stem cells are still poorly understood. In the *X. laevis* intestine (the present study), we found that the expression of Ror2 is specific to the islets consisting of adult stem/progenitor cells, which express mammalian intestinal stem cell markers including Msi1 [17] and LGR5 [18], suggesting the involvement of this non-canonical Wnt pathway in adult stem cell formation. More importantly, our chronological observations indicate that a limited number of cells expressing Ror2, which are scattered in the larval epithelium until stage 59, suddenly change their morphology from simple columnar to islet-like roundish cells at stage 60 and become stained strongly red with MG-PY just as the adult stem/progenitor cells [6]. Thus, Ror2 is the only protein so far whose expression spatiotemporally coincides well with epithelial changes during dedifferentiation into the adult stem cells. This implicates the larval cells expressing Ror2 before metamorphosis as the origin of the adult stem cells in the *X. laevis* intestine.

As for the type of the larval cells expressing Ror2, we morphologically identified them as absorptive epithelial cells possessing the brush border, the major cell type of the intestinal epithelium. This supports our previous proposal that larval differentiated epithelial cells become the adult stem cells through dedifferentiation [26]. In addition, in the present study, the absorptive cells expressing Ror2 were detectable by stage 54 at the latest, when the plasma T3 level is still low [42], and their ratio to total epithelial cells was almost constant during stages 54–59 (our unpublished data). Taken together, it is highly possible that a limited number of larval absorptive cells are predetermined to dedifferentiate into the adult stem cells before metamorphosis, independently of TH, and that Ror2 is useful as the molecular marker to identify such predetermined cells. To verify this possibility, our next step should be directed toward lineage analysis of the epithelial cells expressing Ror2 by using recent frog transgenic technology [45], [46].

### Wnt5a/Ror2 signaling pathway is essential for adult stem cell development

Wnt5a is known to bind directly to Ror2 through its extracellular domain [29]. In the present study, we have shown that the expression of both Wnt5a and Ror2 is indirectly up-regulated by T3 and that Wnt5a protein exists in every tissue except for the degenerating larval epithelium during stages 61–62. Although the spatial expression of its mRNA has not yet been elucidated, recent microarray analysis has shown that the up-regulated expression of Wnt5a is specific to non-epithelial tissues, i.e., the connective tissue or muscles in the *X. laevis* intestine during metamorphosis [25], similar to that in the avian and mammalian guts during organogenesis [47], [48] and the adult mammalian intestine [49]. Thus, it seems likely that Wnt5a protein, which is secreted from the connective tissue or muscles under the influence of T3, at least in part functions as the stem cell niche by binding to Ror2 of the larval epithelial cells.

To clarify the potential roles of the Wnt5a in adult epithelial development, we here investigated the effects of Wnt5a on the *X. laevis* tadpole intestine *in vitro*. In the absence of T3, where endogenous level of Wnt5a protein expression was low, if any, Wnt5a added to the medium caused epithelial morphological changes similar to those during formation of the adult stem cells *in vivo*. However, such changes did not lead to appearance of the adult stem cells expressing Msi1, suggesting that products of T3 response genes other than Wnt5a are necessary for their appearance. This agrees with our previous study showing that adult stem cell formation requires both T3 response genes in the epithelium and those in the non-epithelial tissues [40]. On the other hand, in the presence of T3, where T3 regulates the expression of numerous genes including Wnt5a and induces the adult stem cells [17], the anti-Wnt5a function blocking antibody suppressed the epithelial morphological changes, resulting in the failure of adult stem cell formation. These results indicate that Wnt5a signaling is not sufficient but essential for dedifferentiation of the larval epithelial cells into the adult stem cells. Although precise mechanisms by which Wnt5a affects the epithelial dedifferentiation still remains unclear, Wnt5a/Ror2 signaling is known to transduce the PCP pathway [50] which regulates cell polarity or motility through actin polymerization and microtubule stabilization [29], [51], [52]. Considering that morphological changes caused by Wnt5a were accompanied by changes in cell polarity or motility of the epithelium expressing Ror2 in the present culture study, it is highly possible that Wnt5a plays important roles in the epithelial dedifferentiation by activating the PCP pathway in the larval epithelial cells that express Ror2. Otherwise, as recently reported in human cancers [53], Wnt5a/Ror2 signaling may up-regulate the expression of matrix metalloproteinases (MMPs) such as MMP1, MMP2, and MMP13 to increase cell invasiveness. In fact, among MMPs previously identified as T3 response genes in the *X. laevis* intestine, there is MMP2 whose expression is indirectly up-regulated by T3 and correlates well with adult epithelial development [54]. Although it is still possible that Wnt5a may function via receptor(s) other than Ror2, the involvement of Wnt5a/Ror2 signaling pathway in the stem cell formation well explains why only a limited number of larval epithelial cells can dedifferentiate into the adult stem cells.

As for the other functions of Wnt5a, the present culture study indicated that Wnt5a protein promotes cell proliferation in the *X. laevis* tadpole intestine. However, Wnt5a added to the control medium lacking T3 increased proliferating cells in number, independently of the Ror2 expression. This is different from the case of the T3-treated intestine, where proliferating epithelial cells were strictly localized in the islets expressing Ror2, and suggests that Wnt5a promotes cell proliferation via receptors other than Ror2. In fact, previous microarray analyses reported the expression of several Fzds in the *X. laevis* intestine before and during metamorphosis [23]–[25]. Possibly, different ligand-receptor parings may cause different effects on the *X. laevis* intestine for adult epithelial development.

In conclusion, we have shown here that the absorptive cells expressing Ror2, which is scattered in the larval intestinal epithelium, can dedifferentiate into the adult stem cells in the presence of T3-up-regulated Wnt5a during *X. laevis* metamorphosis and propose that Wnt5a/Ror2 signaling plays a key role in epithelial dedifferentiation by regulating cell polarity or motility. In the human digestive tract, there is a growing body of evidence that mutations or epigenetic changes of Wnt5a and Ror2 are associated with malformations [30] and cancers [53], [55]–[57], although their functions in regulating the adult stem cells remain almost unknown. In addition, in the mouse immature intestine, Ror2 has been reported to be weakly expressed in the intervillus epithelial region [30], where adult stem cells are formed during intestinal maturation when the plasma thyroid hormone levels reach a peak [58], [59] just like during the *X. laevis* intestinal remodeling [60]. Furthermore, in the adult mouse intestine, the expression of both Wnt5 and Fzd2 has been shown to be up-regulated by thyroid hormones in the crypt where the adult stem cells reside [61]. These similarities between the amphibian and mammalian intestines predict an essential function of the thyroid hormone-activated non-canonical Wnt pathway in regulating the intestinal stem cells. It should therefore be interesting from the viewpoints of stem cell and evolutionary biology to further investigate how far roles of Wnt5a/Ror2 signaling in adult stem cell formation are conserved among vertebrates during postembryonic development.

## Supporting Information

Figure S1 **The function-blocking antibody against mouse Wnt5a cross-reacts with** ***X. laevis*** **Wnt5a.** The recombinant mouse Wnt5a (2 ng), *X. laevis* Wnt5a-FLAG, and control IVT samples were subjected to Western blotting. This antibody specifically recognizes the *X. laevis* Wnt5a protein (arrowhead).
(TIF)

Figure S2 **The antibody against human Ror2 cross-reacts with** ***X. laevis*** **Ror2.** FLAG-IP samples prepared from uninjected control and ROR2-FLAG mRNA-injected embryos were subjected to Western blotting with this antibody. A single band observed only in the mRNA-injected sample represents ROR2-FLAG.
(TIF)
