# Supplementary figures and images for: Thyroid Hormone-Regulated Wnt5a/Ror2 Signaling Is Essential for Dedifferentiation of Larval Epithelial Cells into Adult Stem Cells in the Xenopus laevis Intestine
Source: PLoS One. 2014 Sep 11;9(9):e107611. doi: 10.1371/journal.pone.0107611 (PMC4161470; doi:10.1371/journal.pone.0107611)

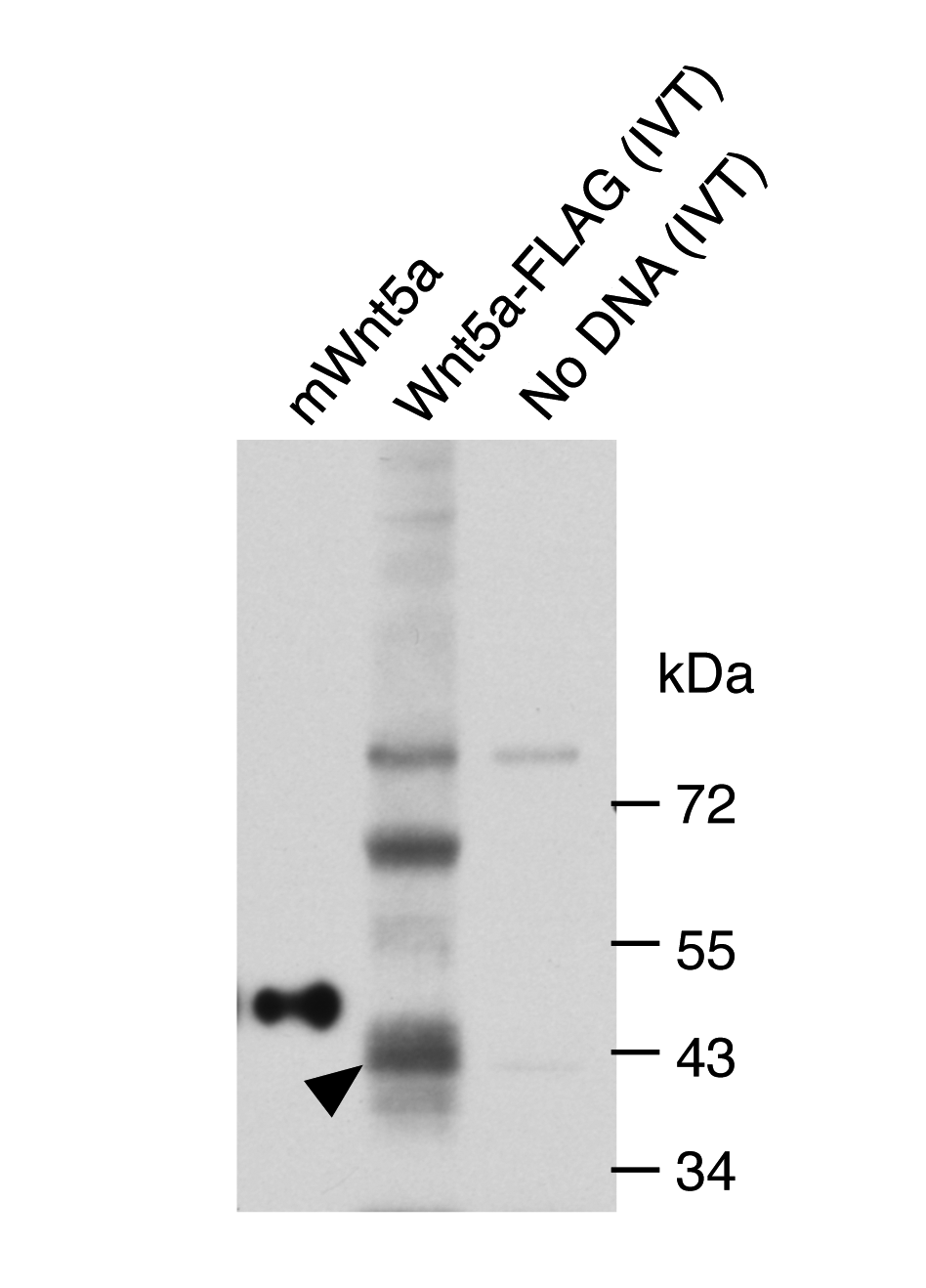

Supplement: Figure S1 — The function-blocking antibody against mouse Wnt5a cross-reacts with X. laevis Wnt5a. The recombinant mouse Wnt5a (2 ng), X. laevis Wnt5a-FLAG, and control IVT samples were subjected to Western blotting. This antibody specifically recognizes the X. laevis Wnt5a protein (arrowhead). (TIF) [file pone.0107611.s001.tif]

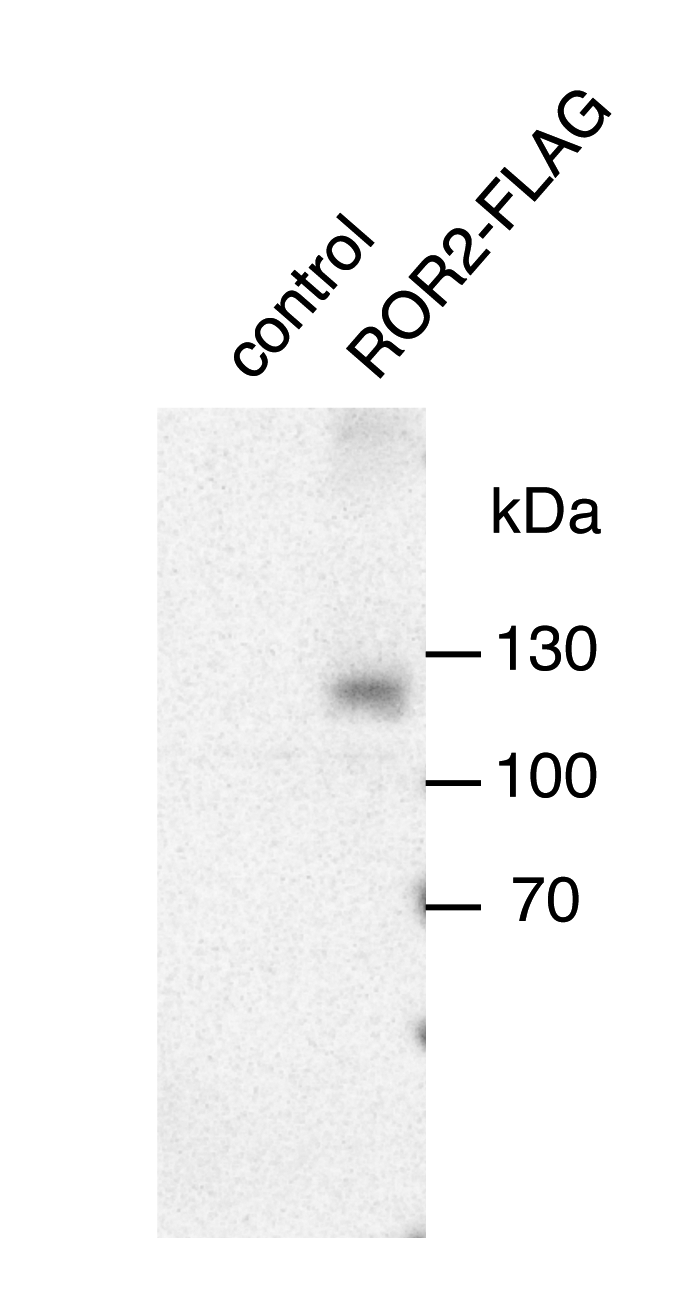

Supplement: Figure S2 — The antibody against human Ror2 cross-reacts with X. laevis Ror2. FLAG-IP samples prepared from uninjected control and ROR2-FLAG mRNA-injected embryos were subjected to Western blotting with this antibody. A single band observed only in the mRNA-injected sample represents ROR2-FLAG. (TIF) [file pone.0107611.s002.tif]
